# Dynactin Subunit p150^Glued^ Is a Neuron-Specific Anti-Catastrophe Factor

**DOI:** 10.1371/journal.pbio.1001611

**Published:** 2013-07-16

**Authors:** Jacob E. Lazarus, Armen J. Moughamian, Mariko K. Tokito, Erika L. F. Holzbaur

**Affiliations:** Department of Physiology and Pennsylvania Muscle Institute, Perelman School of Medicine, University of Pennsylvania, Philadelphia, Pennsylvania, United States of America; Dana-Farber Cancer Institute, United States of America

## Abstract

Regulation of microtubule dynamics in neurons is critical, as defects in the microtubule-based transport of axonal organelles lead to neurodegenerative disease. The microtubule motor cytoplasmic dynein and its partner complex dynactin drive retrograde transport from the distal axon. We have recently shown that the p150^Glued^ subunit of dynactin promotes the initiation of dynein-driven cargo motility from the microtubule plus-end. Because plus end-localized microtubule-associated proteins like p150^Glued^ may also modulate the dynamics of microtubules, we hypothesized that p150^Glued^ might promote cargo initiation by stabilizing the microtubule track. Here, we demonstrate *in vitro* using assembly assays and TIRF microscopy, and in primary neurons using live-cell imaging, that p150^Glued^ is a potent anti-catastrophe factor for microtubules. p150^Glued^ alters microtubule dynamics by binding both to microtubules and to tubulin dimers; both the N-terminal CAP-Gly and basic domains of p150^Glued^ are required in tandem for this activity. p150^Glued^ is alternatively spliced *in vivo*, with the full-length isoform including these two domains expressed primarily in neurons. Accordingly, we find that RNAi of p150^Glued^ in nonpolarized cells does not alter microtubule dynamics, while depletion of p150^Glued^ in neurons leads to a dramatic increase in microtubule catastrophe. Strikingly, a mutation in p150^Glued^ causal for the lethal neurodegenerative disorder Perry syndrome abrogates this anti-catastrophe activity. Thus, we find that dynactin has multiple functions in neurons, both activating dynein-mediated retrograde axonal transport and enhancing microtubule stability through a novel anti-catastrophe mechanism regulated by tissue-specific isoform expression; disruption of either or both of these functions may contribute to neurodegenerative disease.

## Abstract 2

The dynein partner dynactin not only binds to microtubules, but is found to potently influence microtubule dynamics in neurons.

## Author Summary

Microtubules are polymers of tubulin that undergo successive cycles of growth and shrinkage so that the cell can maintain a stable yet adaptable cytoskeleton. In neurons, the microtubule motor protein dynein and its partner complex dynactin drive retrograde transport along microtubules from the distal axon towards the cell body. In addition to binding to dynein, the p150^Glued^ subunit of dynactin independently binds directly to microtubules. We hypothesized that by binding to microtubules, p150^Glued^ might also alter microtubule dynamics. We demonstrate using biochemistry and microscopy *in vitro* and in cells that p150^Glued^ stabilizes microtubules by inhibiting the transition from growth to shrinkage. We show that specific domains of p150^Glued^ encoded by neuronally enriched splice-forms are necessary for this activity. Although depletion of p150^Glued^ in nonpolarized cells does not alter microtubule dynamics, depletion of endogenous p150^Glued^ in neurons leads to dramatic microtubule instability. Strikingly, a mutation in p150^Glued^ known to cause the neurodegenerative disorder Perry syndrome abolishes this activity. In summary, we identified a previously unappreciated function of dynactin in direct regulation of the microtubule cytoskeleton. This activity may enhance generic microtubule stability in the cell, but could be especially important in specific areas of the cell where dynactin and dynein are loaded onto microtubules.

## Introduction

Microtubules are dynamic, polarized polymers of tubulin that serve as tracks for long-distance transport in eukaryotic cells. In neurons, transport along microtubules is especially important yet particularly vulnerable to disruption, as these cells are long-lived and postmitotic with elongated axonal processes that can extend up to a meter [1]. There is accumulating evidence that axonal transport is disrupted in multiple neurodegenerative diseases, including amyotrophic lateral sclerosis and Huntington's disease [2]. In neurodegeneration, defects in microtubule dynamics may precede transport defects [3].

The rate-limiting step in microtubule formation is nucleation from soluble tubulin, which in the canonical pathway is catalyzed by **γ**-TuRC enriched at the centrosome [4]. However, in large, postmitotic cells like neurons, noncentrosomal nucleation may be particularly important [5]–[7]. Following nucleation, the dynamics of polymerization and depolymerization are strongly influenced by microtubule-associated proteins (MAPs). In particular, a spatially specialized group of MAPs that localize to the microtubule plus end, the plus end-tracking proteins (+TIPs), are ideally poised to modulate dynamics in cells [8].

One of these +TIPs is dynactin [9], a large complex that binds and activates cytoplasmic dynein [10],[11] and also associates with microtubules through its dimeric p150^Glued^ subunit [12]. p150^Glued^ has two alternatively spliced microtubule-binding domains at its N-terminus: a cytoskeleton associated protein glycine-rich (CAP-Gly) domain [13], followed by a serine-rich basic domain [14]–[16]. The p150^Glued^ microtubule-binding N-terminus is dispensable for most dynein-mediated organelle transport [16]–[19]. However, we and others have recently shown that it is specifically required for efficient transport initiation from the distal axon in neurons [18],[19].

Because p150^Glued^ is specifically enriched at the microtubule plus end, we hypothesized that it might modify microtubule dynamics. Here, using solution assays and direct visualization of microtubule dynamics using TIRF microscopy, we show that p150^Glued^ promotes microtubule formation by binding both to microtubules and to soluble tubulin. Both the CAP-Gly and basic domains are required for tubulin-binding *in vitro.* The full-length isoform encoding both these domains in tandem is primarily expressed in neurons, so we hypothesized that this pro-polymerization activity might be a neuron-specific function of p150^Glued^. Accordingly, we find that in epithelial cells depleted of p150^Glued^ there is no effect on microtubule dynamics, while in primary neurons, we observed a significant increase in catastrophe upon depletion of p150^Glued^ that was specifically rescued by expression of the neuronal isoform. Finally, we find that a mutation in p150^Glued^ causative for Perry syndrome, a lethal Parkinson's syndrome, inhibits the anti-catastrophe activity. Thus, the novel neuron-specific anti-catastrophe activity described here may facilitate microtubule stabilization in neurons. We speculate that disruption of this function may contribute to neurodegeneration.

## Results

**Figure 1:**
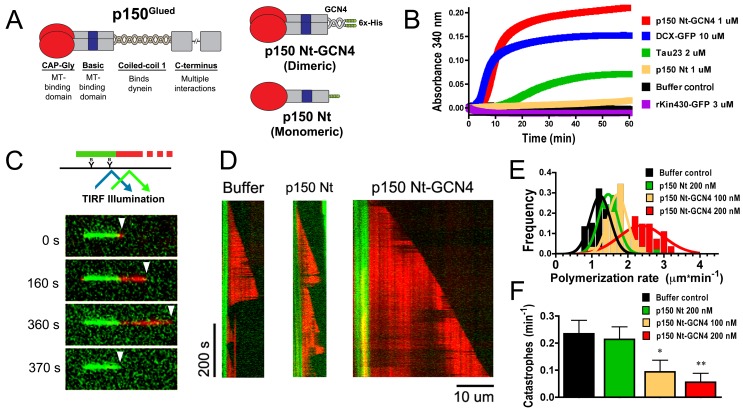
p150^Glued^ promotes microtubule formation. (A) Schematic depicting endogenous full-length p150^Glued^ and dimeric and monomeric constructs. (B) Light scattering traces for recombinant polypeptides or buffer control incubated with tubulin show that the dimeric p150 Nt-GCN4 and neuronal MAPs tau23 and DCX-GFP robustly promote microtubule assembly, while monomeric p150 Nt and rKin430-GFP do not. (C) TIRF elongation assay. As shown in the schematic at top, rhodamine-labeled tubulin is polymerized from biotinylated Alexa488-labeled GMPCPP microtubule seeds. Montage below shows free tubulin assembly (red) from a stabilized seed (green) in the absence of p150. Arrowhead identifies the microtubule plus-end. (D) Kymograph plots showing representative examples with buffer, 200 nM p150 Nt, or p150 Nt-GCN4. (E) Polymerization rates and (F) catastrophe frequencies from seeded assembly shows that p150 Nt-GCN4 promotes polymerization and inhibits catastrophe. All error bars represent SEM of three or more independent experiments. Statistical testing was performed with a two-tailed *t* test. * *p*<0.05; ** *p*<0.01. For (E), *p*<0.01 for all conditions compared to control.

To investigate the dynein-independent effects of dynactin on microtubule dynamics, we designed N-terminal recombinant polypeptides truncated before the dynein binding site within the first p150^Glued^ coiled-coil domain (CC1, Figure 1A) [20]. Since CC1 is also required for endogenous dimerization of p150^Glued^, we replaced this domain with a short, well-characterized GCN4 coiled-coil [21]. In order to assay the effects of dimerization on microtubule dynamics, we also generated a corresponding construct lacking the dimerization domain, but including both the N-terminal CAP-Gly and basic microtubule-binding domains.

To characterize our recombinant polypeptides, we performed glutaraldehyde cross-linking and hydrodynamic analysis, and confirmed that p150 Nt-GCN4 dimerized as expected (Figure S1A). Both the monomeric and dimeric constructs were soluble and monodisperse following purification (Figure S1B). Hydrodynamic analysis also indicated that both polypeptides are highly elongated (R_s_/R_min_>1.9, Figure S1B), consistent with previous electron microscopy images of dynactin [22] that indicate that the p150^Glued^ dimer projects outward from the Arp1 filament that forms the base of the dynactin complex. We measured the relative affinities of both the dimeric and monomeric constructs for microtubules, and found that both bound to paclitaxel-stabilized microtubules with moderate affinities (K_d_ of 340 nM and 890 nM, respectively, Figure S1C), within the typical range for CAP-Gly proteins [23].

### p150^Glued^ Dimer Potently Promotes Microtubule Formation

Previous work from our lab suggested that the N-terminus of p150^Glued^ promotes bulk microtubule formation [24]. To confirm and extend this result, we first compared the activities of the dimeric p150 Nt-GCN4 and the monomeric p150 Nt constructs using in vitro assembly assays. Only the dimeric construct induced a large increase in light scattering, a measure of increased microtubule polymerization (Figure 1B).

We also compared the activities of these constructs to that of the well-characterized neuronal MAPs tau (Tau23) and doublecortin (DCX-GFP) (Figures 1B and S1D), both of which have been shown to promote the formation of microtubules, a function that appears defective in the setting of disease [25],[26]. The increase in light scattering induced by 1 µM p150 Nt-GCN4 was similar in magnitude to the increase induced by addition of 10 µM DCX-GFP, and approximately 4-fold more than the increase induced by 2 µM Tau23 (Figure 1B). Thus, p150, like these classical neuronal MAPs, enhances the polymerization of microtubules from soluble tubulin. In contrast, the microtubule-binding motor domain of kinesin-1 (rKin430-GFP) did not promote the formation of microtubules (Figure 1B), indicating that this effect is not a nonspecific one characteristic of all microtubule-binding proteins.

Since microtubule bundling, as well as microtubule polymerization, can lead to increases in light scattering signal, we pelleted the reaction mixtures and analyzed the resulting microtubule pellets by SDS-PAGE (Figure S1E). This analysis confirmed that p150 Nt-GCN4 induces a large increase in tubulin polymerization. Additionally, we tested to ensure that increased turbidity from protein aggregation was not responsible for the light scattering signal; when the purified p150Nt-GCN4 is incubated in the absence of tubulin, light scattering remained low and constant (Figure S1F). As a final control, because the fusion of a His-affinity tag has been reported to disrupt the activity of another plus-tip protein, EB1 [27], we compared our His-tagged p150 constructs to equivalent Strep-tagged constructs, as well as to the activity of an untagged construct purified by ion exchange chromatography (Figure S2A). The untagged construct was identical to the His-tagged construct on size exclusion chromatography (Figure S2B), and the activities we measured were identical (Figure S2C, S2D), indicating that the promotion of microtubule polymerization by dimeric p150 is not affected by the nature of the N-terminal purification tag.

Though previous work concluded that monomeric p150 constructs could influence microtubule polymerization at high concentrations [28],[29], in our assay, monomeric p150 Nt only marginally increased light scattering as compared to dimeric p150 Nt-GCN4 (Figure 1B); nor did we see increased tubulin in polymer in sedimentation assays as compared to buffer controls (Figure S1E). Thus, we find that dimerization of p150^Glued^ is required for robust pro-polymerization activity in vitro.

### p150^Glued^ Enhances Microtubule Polymerization Rates and Inhibits Microtubule Catastrophe

We hypothesized that p150^Glued^, which localizes to microtubule plus-ends in the cell [9],[16], might, like other +TIPS [8], influence the parameters of dynamic instability. In the absence of MAPs, microtubule dynamic instability is characterized by periods of slow polymer growth ending in catastrophe and rapid depolymerization; following rescue, microtubule growth resumes by addition of new tubulin subunits at the plus end. To test the hypothesis that p150^Glued^ alters these parameters, we used total internal reflection fluorescence (TIRF) microscopy to directly observe microtubule polymerization from pre-formed GMPCPP-stabilized microtubule seeds (Figure 1C) [30],[31].

Addition of tubulin alone to pre-formed, stabilized seeds induced characteristic periods of slow growth, with transitions to rapid shrinkage following a catastrophe (Figure 1D). In contrast, addition of a low (100–200 nM), physiological concentration of p150 Nt-GCN4 [32] to the assay led to more rapid microtubule growth (Figure 1D). The polymerization rate increased more than 2-fold at 200 nM p150 Nt-GCN4 (*p*<0.01, Figure 1E). We also observed that growth was more persistent, with the catastrophe frequency reduced 2-fold at 100 nM and 4-fold at 200 nM (*p*<0.05 and <0.01, Figure 1F).

In agreement with the results from the light scattering assay described above, here by direct observation we also see that dimerization of p150^Glued^ is necessary to influence microtubules. The monomeric p150 Nt construct had only a modest effect on microtubule polymerization (Figure 1E), and did not affect the catastrophe frequency (*p*>0.8, Figure 1F), while dimeric p150^Glued^ had a robust effect on microtubule dynamics, acting to both enhance the polymerization rate and suppress the catastrophe frequency *in vitro.*

### p150^Glued^ Modifies Microtubule Dynamics Independently of EB1

Previous studies with a monomeric p150^Glued^ CAP-Gly polypeptide lacking the basic domain suggested that EB1 was required for p150^Glued^ to modify microtubule dynamics [28],[29]. However, native p150^Glued^ is dimeric, and our dimeric p150 Nt-GCN4 construct has potent effects on microtubule dynamics in the absence of EB1. However, we wondered if the addition of EB1 to our assays might further modulate the effects we observed.

Using analytical size exclusion chromatography, we confirmed that, as expected [28],[33],[34], p150 Nt-GCN4 forms a stable complex with recombinant full-length EB1 (Figure S3A–B). However, the ability of p150^Glued^ to promote microtubule formation appears to be independent of this interaction; the addition of EB1 caused a minor decrease on the pro-polymerization activity of p150^Glued^ (Figure S3C, S3E) and did not affect its ability to inhibit microtubule catastrophe (*p*>0.5, Figure S3D, S3F).

These data confirm that p150^Glued^ has an intrinsic ability to modify microtubule dynamics and, further, that p150^Glued^ inhibits the inherent pro-catastrophe effects of EB1 *in vitro*.

### p150^Glued^ Catalyzes Microtubule Nucleation

**Figure 2:**
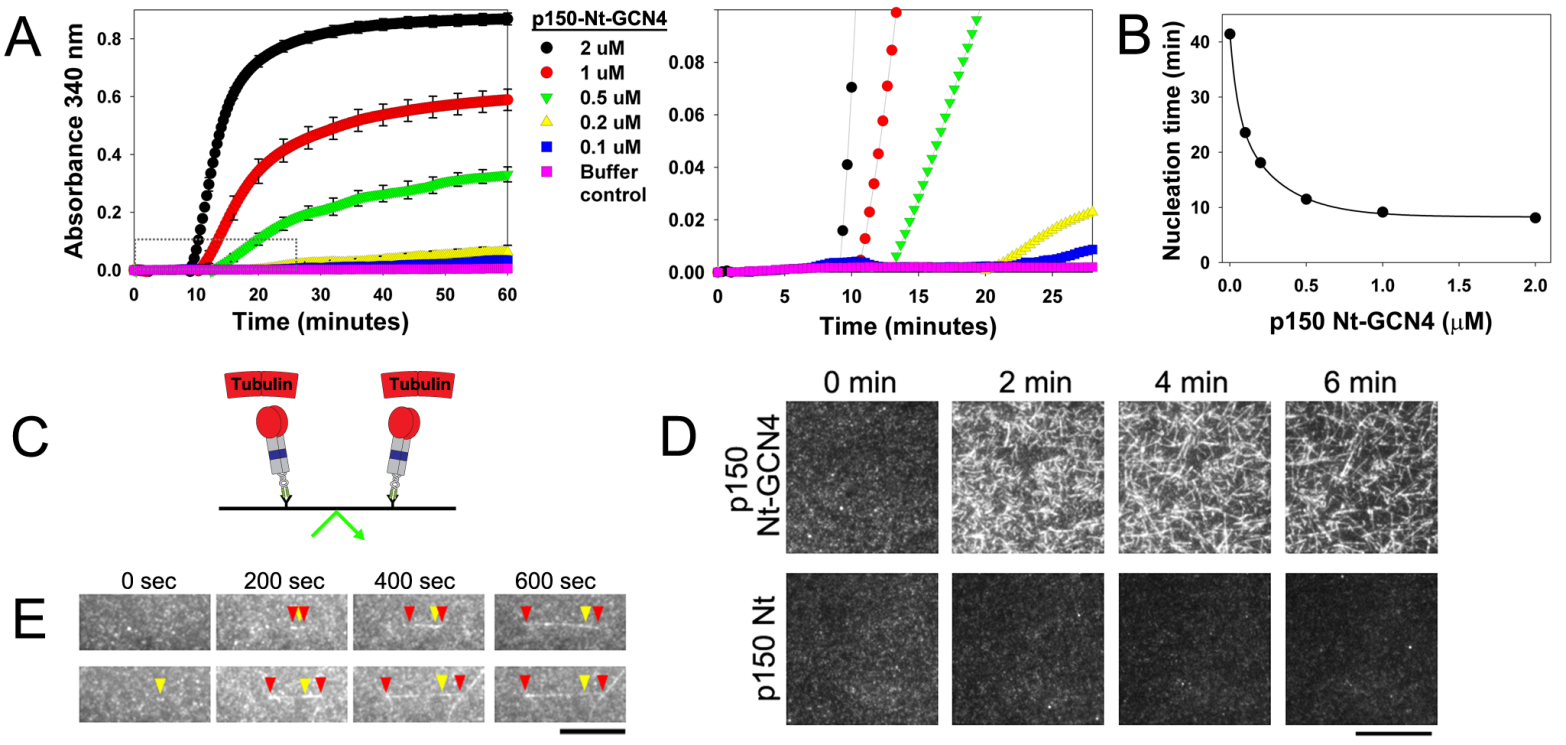
p150 catalyzes microtubules nucleation. (A) Light scattering traces for increasing concentrations of p150 Nt-GCN4. At right, the scale was magnified to illustrate the initial increase in light scattering observed with increasing concentrations of p150 Nt-GCN4. Error bars represent SEM of three or more independent experiments and are omitted for clarity at right. (B) p150 Nt-GCN4 decreases nucleation time, defined as initial appearance of signal over background. Fit is to a double exponential decay. Note that error bars are smaller than symbols. (C) Schematic of the TIRF nucleation assay. p150 constructs are immobilized and the chamber is then perfused with rhodamine-labeled tubulin. (D) Montage from TIRF nucleation assay shows that dimeric p150 Nt-GCN4 catalyzes microtubule nucleation in contrast to monomeric p150 Nt. Scale bar, 12.5 µm. (E) Montage of representative microtubules nucleated in the presence of a low concentration of p150 dimer. Yellow arrows identify the site of nucleation. Red arrows identify the growing plus- and minus-ends. Scale bar, 5 µm.

We have shown that p150 Nt-GCN4 acts as a pro-polymerization and anti-catastrophe factor *in vitro*, acting on preformed microtubules. However, we suspected that p150 Nt-GCN4 might also promote microtubule nucleation because we observed a decrease in the lag time for microtubule formation that was inversely dependent on the p150 Nt-GCN4 concentration (Figure 2A, 2B).

We designed a TIRF assay to directly test this hypothesis. We specifically oriented the p150 N-terminal microtubule-binding domains toward the microscopy chamber by immobilizing 250 nM p150 polypeptides on the coverslip via an antibody to the C-terminal His-tag (Figure 2C). We then washed the chamber to remove unbound p150, and perfused in 3.5 µM soluble tubulin, which is below the critical concentration for spontaneous microtubule nucleation.

Consistent with our hypothesis, in the presence of dimeric p150 Nt-GCN4, we observed the appearance of many short microtubules within 2 min (Figure 2D). We did not observe nucleation when monomeric p150 Nt was immobilized in the chamber (Figure 2D). When we reduced the amount of p150 Nt-GCN4 on the chamber surface so that we could observe the nucleation of individual filaments, we observed growth from both the microtubule minus- and plus-ends (Figure 2E). This suggested to us that p150 was not promoting nucleation via a template-and-capping mode like γ-TuRC [4], but instead might stabilize an oligomeric tubulin species, similar to the action of doublecortin [6], or by binding directly to soluble tubulin dimers.

### p150^Glued^ Binds to Soluble Tubulin Dimers

p150^Glued^ is well-characterized as a MAP, but we hypothesized that it might affect microtubule dynamics by also binding to soluble tubulin through its CAP-Gly domain. The CAP-Gly domains of other structurally related proteins, such as CLIP-170 and tubulin binding cofactors B and E, have been shown to bind soluble tubulin [23]. We used analytical size exclusion chromatography to assay complex formation. To preclude microtubule nucleation, we performed all experiments at 4 C.

**Figure 3:**
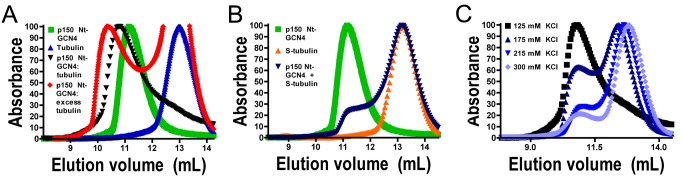
p150^Glued^ forms a complex with soluble tubulin. (A) Size exclusion chromatograms for p150 Nt-GCN4 run alone or pre-incubated with tubulin reveal that p150 forms a stable complex with tubulin. For the red trace, maximum absorbance is scaled to the complex peak. (B) Size exclusion chromatograms for subtilisin-treated tubulin incubated with p150 Nt-GCN4 suggest that the p150^Glued^ binds tubulin C-termini in solution, as complex formation is not observed after cleavage. (C) Size exclusion chromatograms for p150 Nt-GCN4 incubated with tubulin and eluted at increasing ionic strength indicate that electrostatic interactions between tubulin and the p150 basic domain stabilize the p150-tubulin complex because progressively reduced complex formation is observed.

When both p150 Nt-GCN4 and p150 Nt were incubated with tubulin, we observed a pronounced shift in the elution peak, indicating complex formation (Figure 3A, Figure S4A–B). We varied the molar ratio of p150∶tubulin until we no longer observed uncomplexed species [35]. In this way, we found that p150 Nt-GCN4 formed a stable complex with tubulin in a 1∶1 ratio (Figure S4C,E–F). However, we observed a tail trailing the elution peak of the 1∶1 p150 Nt-GCN4∶tubulin complex (Figure 3A), which could indicate an exchange process between free p150 Nt-GCN4 and tubulin during the elution. In agreement with this possibility, we found that when we incubated excess tubulin with p150 Nt-GCN4 to encourage saturated binding, the elution peak shifted further to the left (Figure 3A, Figure S4H–I), indicating that a larger complex had formed, and suggesting that p150 Nt-GCN4 might promote microtubule nucleation by transiently binding multiple tubulin subunits and encouraging the formation of a stable seed.

We know that the acidic C-terminal domain of tubulin is important in the binding of p150^Glued^ to microtubules [33],[36]. To determine if p150^Glued^ also binds to soluble tubulin dimers via their C-terminal tails, we cleaved these tails from tubulin using the protease subtilisin (Figure S4J–K) [37],[38] and again assayed for complex formation via size exclusion chromatography. After cleavage, we could not detect a tubulin-p150 complex (Figure 3B), confirming that the p150 CAP-Gly domain interacts with the C-terminus of tubulin dimers in solution.

This orientation might leave the p150 basic domain free to stabilize the complex through electrostatic interactions. In support of this model, we found that when we eluted the tubulin-p150 Nt-GCN4 complex under increasing ionic strength, which should disrupt such interactions, the relative abundance of the complex was decreased at the expense of free tubulin (Figure 3C). This suggests that while either the CAP-Gly or the basic domains are sufficient to bind microtubules [14]–[16], both domains may be necessary for p150^Glued^ to bind tubulin dimers and promote microtubule assembly.

### Neuron-Specific p150^Glued^ Isoform Stabilizes Microtubules

**Figure 4:**
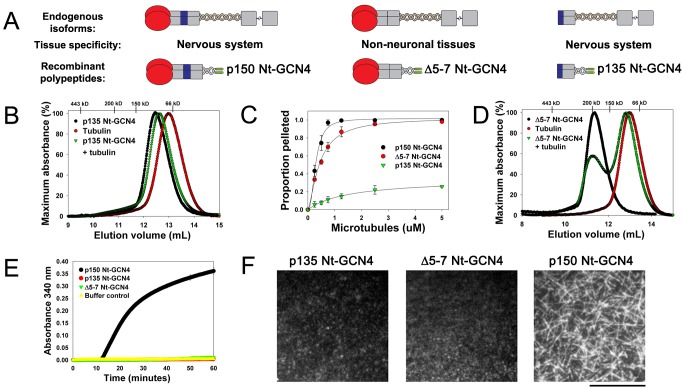
Tissue-specific p150^Glued^ isoforms differentially modify microtubule assembly dynamics. (A) Schematic depicting p150^Glued^ spliceforms (and corresponding recombinant proteins): the neuronally-enriched p150 and p135 (p135 Nt-GCN4), and the ubiquitous isoform lacking the basic region (exons 5–7, Δ5–7 Nt-GCN4). (B, D) Size exclusion chromatography with (B) p135 Nt-GCN4 or (D) Δ5–7 Nt-GCN4 indicates that they cannot form stable complexes with tubulin. (C) Microtubule affinity of 400 nM dimeric polypeptides for increasing concentrations of Taxol-stabilized microtubules demonstrates that these constructs bind microtubules. (E) Light scattering traces for 1 µM constructs suggest that only the brain-specific full-length p150 isoform can promote microtubule formation. Error bars (± SEM) are smaller than the symbols. (F) Single frames from 6 min posttubulin perfusion contrasting the inability of Δ5–7 Nt-GCN4 or p135 Nt-GCN4 to nucleate microtubules with the full-length p150 Nt-GCN4. Scale bar, 12.5 µm.

p150^Glued^ is alternatively spliced in a tissue-specific manner with expression of full-length p150 containing both the CAP-Gly and basic domains restricted to the nervous system (Figure 4A) [15],[16]. The two other predominant p150 spliceforms expressed *in vivo* lack either the CAP-Gly domain or the basic domain. p135 is an isoform that, like p150, is enriched in the nervous system; it arises from an alternative start site and thus lacks the CAP-Gly domain and exon 5 [39]. In nonneuronal tissues, all or part of the basic domain is spliced out [15],[16]. Thus we were able to use physiologically relevant splice forms to test our model.

We produced recombinant dimeric polypeptides recapitulating p135 (p135 Nt-GCN4) and p150 lacking exons 5, 6, and 7 (the majority of the basic region, Δ5–7 Nt-GCN4) (Figure 4A and Figure S5A–D). We found that when we co-incubated p135 Nt-GCN4 (which lacks the CAP-Gly domain) with tubulin, we did not observe complex formation by size exclusion chromatography (Figure 4B). This was unsurprising given the accepted role of CAP-Gly domains in binding to microtubules [23], and the role we establish here for p150-binding to tubulin dimers in solution. We next assayed Δ5–7 Nt-GCN4, which lacks only the 23 amino acid residue basic domain; deletion of this domain only marginally impacts microtubule-binding affinity as measured by co-pelleting (K_d_ = 470 nM versus 290 nM for p150 Nt-GCN4, Figure 4C) [14],[16], and does not affect EB1 binding as assessed by size exclusion chromatography (Figure S5E–F) [23]. Strikingly, we find that this isoform cannot bind tubulin (Figure 4D). Further, neither p135 Nt-GCN4 nor Δ5–7 Nt-GCN4 were able to promote the formation of microtubules as assessed both by light scattering and direct TIRF imaging (Figure 4E, Figure 4F). These results demonstrate that both the tandem p150^Glued^ CAP-Gly and basic domains are necessary to stably complex tubulin and to promote assembly dynamics.

### p150^Glued^ Is a Tissue-Specific Microtubule Modifier

**Figure 5:**
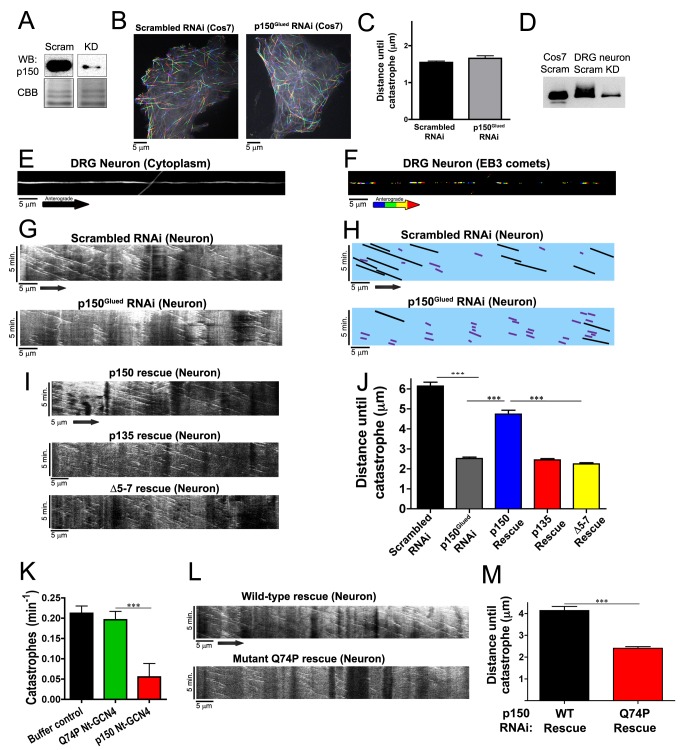
p150^Glued^ stabilizes microtubules in neurons. (A) Depletion of p150^Glued^ in COS7 cells by siRNA (KD) relative to control cells treated with scrambled (Scram) oligonucleotides; upper panel is a Western blot probed with a monoclonal antibody to p150^Glued^, and lower panel is Coomassie staining to show equal protein loading. (B) Representative rainbow-coded maximum intensity projections show that the overall microtubule architecture and dynamics are not perturbed in COS7 cells depleted of p150^Glued^ relative to control cells. (C) PlusTipTracker quantitation of EB3-GFP comet velocities shows that knockdown does not alter parameters of microtubule dynamics, including distance to catastrophe, a measure of the catastrophe frequency (*p*>0.1). (D) Western blot showing knockdown of p150^Glued^ in DRG neurons (KD), relative to control neurons or COS7 cells treated with scrambled oligonucleotide. Note the differential splice forms of p150^Glued^ expressed in neurons relative to COS7 cells. (E) Space-filling fluorescence in representative neurites used to investigate microtubule dynamics in primary DRG neurons. (F) Rainbow-coded maximum intensity projections of selected EB3-GFP comets demonstrate microtubule dynamics in DRG neurons. (G) Kymographs of GFP-EB3 comets in cultured DRG neurons treated with either scrambled or p150^Glued^ RNAi reveal that in neurons, p150^Glued^ inhibits catastrophe. (H) Camera lucida tracing of the kymographs in panel G to indicate polymerization events persisting greater than 5 µm (black) and less than 2 µm (magenta). (I) Kymographs of GFP-EB3 comets in neurons depleted of endogenous p150^Glued^ by RNAi and rescued with either full-length p150, Δ5–7, or the neuron-specific alternative splice form p135. (J) Analysis of microtubule dynamics in scrambled control neurons, and neurons depleted of endogenous p150^Glued^ with or without rescue with resistant constructs of p150 or p135 and Δ5–7. Bars represent mean of comet parameters from multiple cells on multiple days ± SEM. Statistical testing was performed via *t* test with correction for multiple comparisons. *** *p*<0.001. (K) In vitro analysis of catastrophe rates demonstrates that the Perry syndrome-associated mutation Q74P p150 Nt-GCN4 does not inhibit microtubule catastrophe (*p*>0.5), as compared to the wild-type p150Nt-GCN4 construct. (L, M) Kymographs and quantitation of GFP-EB3 comets in cultured DRG neurons depleted of endogenous p150^Glued^ and rescued with either wild-type or Q74P p150^Glued^ reveal that mutant p150 is defective in inhibiting microtubule catastrophe in neurons.

To determine how the cellular role of p150^Glued^ corresponds to the biochemical activities we measured, we first depleted p150^Glued^ from COS7 cells (Figure 5A), a primate cell line derived from kidney that should not express significant levels of the neuronal isoform [15]. We transfected the cells with low levels of GFP-EB3 to visualize the growing microtubule plus end, and examined microtubule dynamics in an unbiased manner using the PlusTipTracker software package [40]. Under conditions in which >95% of endogenous p150^Glued^ was depleted (Figure 5A), we did not observe an appreciable effect on either the average displacement of EB3-GFP comets before microtubule catastrophe or on microtubule polymerization velocities (Figure 5B–C, *p*>0.1, and unpublished data). We obtained similar results in HeLa cells, another epithelial cell line.

We next investigated the effect of p150^Glued^ depletion on microtubule dynamics in mammalian neurons, which express the full-length isoform which includes both the CAP-Gly and basic domains that we have identified as necessary for productive tubulin binding. Interestingly, though the CAP-Gly domain is a conserved feature of p150^Glued^, the basic domain appears to be specifically evolved in organisms with complex, dynamic microtubule cytoskeletons. It is absent in yeasts, but is apparent in protists, filamentous fungi, and vertebrates, with mammalian taxa expressing particularly basic regions downstream of the CAP-Gly domain (Figure S6A–B, Dataset S1) [41].

Next, we visualized microtubule dynamics using GFP-EB3 expressed at low levels in mouse dorsal root ganglion neurons (DRGs; Figure 5E,F). Because of the high levels of tubulin in mammalian neurons, we quantitated microtubule dynamics manually using kymograph analysis (Figure 5G). When p150^Glued^ was depleted by ∼80% (Figure 5D), we did not observe significant differences in either polymerization rates or the number of EB3-GFP comets compared to scrambled RNAi-treated cells (unpublished data). However, we noted that when p150^Glued^ was depleted, there was a significant decrease in the distance GFP-EB3 comets traveled before catastrophe (Figure 5G,H), indicating a significant increase in the catastrophe frequency (Figure 5J). For example, compare the comets that traveled at least 5 µm before catastrophe (black) compared to those that travelled less than 2 µm (magenta) as shown in Figure 5H.

To confirm that this was a specific effect of p150^Glued^ knockdown, we quantitated dynamics in RNAi-treated neurons rescued with either the full-length neuronal p150 isoform, or with p135, which lacks the N-terminal CAP-Gly domain. We observed that rescue of neurons with a plasmid encoding RNA-resistant p150 significantly reduced the catastrophe frequency as compared to RNAi-treated cells (Figure 5I,J). Wild-type dynamics were not completely restored, likely because we did not fully restore endogenous expression levels with the rescue plasmid (Figure S6C). In contrast, transfection with the p135 isoform, which lacks the N-terminal CAP-Gly domain, did not rescue defective polymerization dynamics in primary neurons (Figure 5I,J). Importantly, we found that transfection with the Δ5–7 construct, the nonneuronal splice form that lacks the basic domain, also did not rescue the defective polymerization dynamics induced by depletion of endogenous p150^Glued^ (Figure 5I,J). Thus, in strong agreement with our *in vitro* results, these observations indicate that the tandem CAP-Gly and basic domains of p150 are required to generate anti-catastrophe activity in neurons.

### A Parkinson Disease Mutation Abolishes p150^Glued^ Activity

Mutations in the CAP-Gly domain of p150^Glued^ have been found to cause multiple neurodegenerative diseases, including Perry syndrome, a disease characterized by Parkinsonism, weight loss, hypoventilation, and depression. While human disease-associated mutations in p150^Glued^ are expressed throughout the body, these mutations induce pathologies only in the nervous system [2]. Thus, we hypothesized that the tissue-specific activity of p150^Glued^ described above might be disrupted in this disease.

To test this hypothesis, we focused on the Q74P mutation (Figure S7A) [42]. The Q74P mutation has been shown to disrupt both the microtubule and EB1-binding activities of the p150 CAP-Gly domain but to have only a modest effect on overall protein stability [19],[43]. To obviate any disease-induced aggregation, we expressed and purified recombinant Q74P Nt-GCN4 immediately before experimentation using a final gel filtration step to exclude higher order oligomers (Figure S7B, S7C), and confirmed proper dimerization by glutaraldehyde cross-linking (Figure S7D). In contrast to wild-type p150 Nt-GCN4, we found that Q74P Nt-GCN4 was defective in inhibiting microtubule catastrophe *in vitro* (Figure 5K). In primary neurons, we found that transfection with a plasmid expressing Q74P could not restore normal microtubule dynamics, in contrast to the rescue seen with the wild-type p150^Glued^ construct (Figure 5L,M).

## Discussion

**Figure 6:**
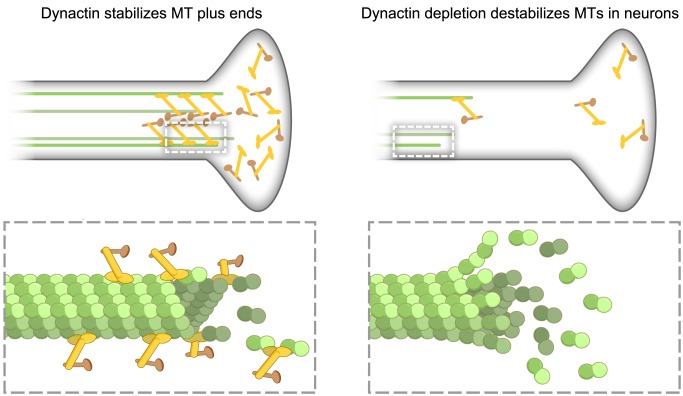
p150^Glued^ is a neuron-specific microtubule anti-catastrophe factor. p150^Glued^ inhibits microtubule catastrophe by binding both to microtubules and to soluble tubulin, leading to enhanced microtubule stability along the axon. Depletion of endogenous p150^Glued^ by RNAi leads to more frequent microtubule catastrophe in neurons.

Here we show that p150^Glued^ promotes microtubule formation *in vitro* by catalyzing nucleation, increasing the polymerization rate, and inhibiting catastrophe. These activities require dimerization and are dependent on the ability of p150^Glued^ to form a stable complex with tubulin through interactions with both the N-terminal CAP-Gly and basic domains. In primary neurons, we observe that the dominant effect of p150^Glued^ on microtubule dynamics is the suppression of catastrophe (Figure 6). Finally, we determine that a single point mutation within the CAP-Gly domain of p150^Glued^ causative for a fatal familial form of Parkinson disease, known as Perry Syndrome, leaves p150^Glued^ unable to promote microtubule assembly either *in vitro* or in neurons.

Dynactin was originally identified as a large protein complex that supported dynein-mediated vesicle transport [32],[44]. The N-terminus of the 150 kDa subunit binds microtubules independently of dynein [12], and increases the processivity of dynein *in vitro* [14],[45]. Recently, it has been demonstrated that the microtubule-binding N-terminus of p150^Glued^ is dispensable for organelle localization and vesicular motility in nonneuronal cells [16],[17],[19]. However, the microtubule-binding CAP-Gly domain of dynactin is required for efficient transport initiation from the distal axon in neurons [18],[19]. A plus end-localized pool of p150^Glued^ may serve to load dynein onto the microtubule [19],[46]. However, biochemical analyses and immunolocalization suggest that a large proportion of dynactin may in fact not be in complex with dynein [19],[32],[47],[48]. Enriched at the plus end, this population of dynactin would be perfectly poised to affect microtubule dynamics.

Our data suggest a mechanism whereby p150^Glued^ could modify microtubule dynamics. Recently, it has been suggested that the kinetics of tubulin association and dissociation with the microtubule plus end may be much faster than previously appreciated [49]. This makes it increasingly plausible that one mode whereby MAPs alter microtubule dynamics is by modulating the off-rate of tubulin subunits from microtubule plus ends. Since p150^Glued^ can bind both to microtubules and to soluble tubulin, and because dimerization appears necessary for p150^Glued^ to robustly modify dynamics, we speculate that p150^Glued^ may be acting in this capacity by binding to both microtubules and tubulin at the same time, decreasing the off-rate and inhibiting catastrophe, enabling efficient initiation of dynein-mediated retrograde runs (Figure 6). Interestingly, this mechanism is distinct from the mode by which cytoplasmic dynein independently functions to inhibit catastrophe [50]. Areas of the distal neuron where both cytoplasmic and dynactin are localized could be sites of particularly robust microtubule stabilization.

The regulation of these microtubule-modifying abilities of p150^Glued^ may be multifactorial. We have shown that the basic region is necessary for the modification of microtubule dynamics by p150^Glued^, likely by ensuring a stable complex with the distributed acidic nature of tubulin. The basic region is also serine- and threonine-rich, and has been shown to be the target of phosphorylation by regulatory kinases [51],[52], which might further modulate the p150-tubulin interaction during mitosis, or during development. p150^Glued^ also binds to CLIP-170 [24],[28],[34],[53], which could further modify the behavior of p150^Glued^ in the cell.

*In vivo*, only the p150^Glued^ isoform expressed in neurons includes both the full CAP-Gly and basic domains that we have shown are necessary to modify microtubule assembly dynamics. We have recently shown that young, developing neurons depleted of p150^Glued^ are morphologically normal [19]. In fact, profound depletion of both EB1 and EB3, which should effectively disrupt plus-end targeting, has no gross effects on neurite outgrowth [19]. It may be that, as recent evidence suggests, only as neurons age, and their processes lengthen and elaborate, does the centrosome lose its function as a microtubule organizing center and microtubule dynamics become particularly reliant on plus-end regulation [5],[7]. It is perhaps telling that human patients with the Q74P p150^Glued^ mutation do not show disease onset until the fifth decade of life [42]. More broadly, microtubule dynamics may alter in aging or degenerating neurons, as suggested from studies of cells from patients with sporadic Parkinson's and Alzheimer's disease [54]–[56].

In summary, we have identified and characterized a novel role for p150^Glued^ in the tissue-specific stabilization of microtubules, and implicated defects in neurodegeneration. Further studies to disentangle the effects of the mutation on axonal transport and microtubule stability in neurons will be required to clarify the pathogenesis involved.

## Experimental Procedures

### Protein Purification

Amino acids 1–210 of human p150^Glued^ (DCTN1, NM_004082.4) were inserted into pET-29a (Novagen) with a short linker (AAAADPPVAT) before the C-terminal 6×-His tag. Dimeric constructs contained a GCN4 sequence (VMKQLEDKVEELLSKNYHLENEVARLKKLVGE) [21] before the linker and 6×-His. Δ5–7 dimer was deleted for exons 5–7 (RGLKPKKAPTARKTTTRRPK). The p135 dimer encodes an N-terminal MMRQ (replicating the endogenous expressed spliceform) appended to p150^Glued^ AA 139–210. Q74P Nt-GCN4 was constructed from the p150 Nt-GCN4 backbone and previously used plasmids [19]. The tag-less p150^Glued^ construct was similar to 1–210-GCN-His, but lacked the C-terminal His-tag. The Strep-tagged construct was made by replacing the C-terminal His-tag by PCR. C-terminal GFP-tagged constructs were expressed from pET-29a with EGFP-6×-His appended after the above short linker. The accuracy of all inserts was verified by sequencing.

For expression, transformed Rosetta *E. coli* (Novagen) were grown in LB at 37°C and 275 rpm under standard chloramphenicol and kanamycin selection. At OD_600_ of ∼0.8, cultures were cooled to 15°C and induced with 0.5 mM IPTG. After 16 h, cells were harvested and resuspended in binding buffer (500 mM NaCl, 20 mM Tris-HCl pH 7.9, 20 mM imidazole, 1 mM tris(2-carboxyethyl)phosphine, supplemented with complete protease inhibitor cocktail from Sigma, TAME, leupeptin, and PMSF). At that point, cells were either flash-frozen in liquid nitrogen, or lysed via French press (Thermo) at 18,000 psi. Nucleic acids were cleaved with DNAse I and RNAse H treatment (Roche) and the lysate was cleared at 45,000 g for 20 min at 4°C. The supernatant was filtered through a 0.22 µm polysulfone membrane (PALL Life Sciences) and applied to a 2 mL V_b_ Ni Sepharose Fast Flow column by gravity or a 1 mL HisTrap column (GE Healthcare) by FPLC. To purify the Strep-tagged construct, a 1 mL Strep-Tactin Superflow Plus column (Qiagen) was used, and for the un-tagged construct, this step was omitted. The column was washed with binding buffer until the UV absorbance had settled, then eluted with binding buffer plus 0.5 M imidazole (or desthiobiotin for Strep-tagged construct). Protein-containing fractions were then diluted in 100 mM NaCl binding buffer, loaded into a 50 mL Superloop (GE Healthcare), and injected onto a Mono S 5/50 GL column (GE Healthcare), and eluted with a linear gradient of 0.1 M NaCl to 1 M NaCl in binding buffer. Finally, the protein was exchanged into BRB80 (80 mM PIPES pH 6.8, 1 mM MgCl_2_, 1 mM EGTA) with 75 mM KCl and 10% glycerol (v/v) by gel filtration on a Superdex 200 10/300 GL column (GE Healthcare), aliquoted, and flash-frozen and stored in liquid N_2_. Protein concentration was determined by BCA assay (Pierce) with BSA as a standard. We found that for the p150 constructs, concentration determined in this way differed by only ∼10% when compared to concentration determined spectrophotometrically with a calculated extinction coefficient. All concentrations are expressed assuming either complete or absent dimerization, as appropriate from experimentation or the literature.

Unlabeled tubulin was purified from bovine brain through two cycles of polymerization and depolymerization in high-molarity PIPES buffer as described [57]. Tau23 was purified as previously reported [58]. EB1 was purified as previously reported [31], except 0.5 M NaCl and 10% glycerol were present throughout, and protein was gel-filtered as for p150 constructs before aliquotting. DCX-GFP and rKin430-GFP were purified after Bechstedt and Brouhard [26].

### Glutaraldehyde Cross-Linking

Dimeric constructs were diluted to 0.5 µM and the p150 monomer was diluted to 1 µM in BRB20 with 125 mM NaCl and 1 mM DTT and incubated on ice with 0.5% glutaraldehyde (Thermo) for 2 h. The reactions were quenched with an equal volume of 1 M glycine, denatured, and analyzed by SDS-PAGE and colloidal Coomassie staining (Invitrogen).

### Analytical Size Exclusion Chromatography

Recombinant polypeptides were thawed and incubated at the molar ratio indicated in the text with 30 µM tubulin or mock-incubated for 10′ on ice, then loaded into a 50 µL loop with 80 µL final volume, and injected onto a Superdex 200 10/300 GL column pre-equilibrated with BRB20 (pH 7.1) supplemented with 125 mM KCl and 10 mM imidazole, or indicated concentrations of KCl for Figure 3C. Polypeptides were eluted isocratically at 4°C and 0.4 mL/min in the equilibration buffer and collected in 250 µL fractions. The column was calibrated with gel filtration molecular weight standards (ferritin, β-amylase, alcohol dehydrogenase, bovine serum albumin, and carbonic anhydrase) from Sigma. The void volume was at ∼7.5 mL. We did not detect any material in the void for any experiments.

Subtilisin cleavage was performed for the indicated times at room temperature in a 1∶350 mass ratio to tubulin after Knipling et al. [38] and Gupta et al. [59].

### Light Scattering Assay

Assay was performed in BRB80 supplemented with 35 mM KCl, 5% glycerol (v/v), 1 mM DTT, 1 mM GTP, and 1 mg/mL casein (to prevent nonspecific adsorption) mixed with 20 µM tubulin in a 96-well half area UV transparent plate (Corning) on ice. The plate was read in kinetic absorption mode at 340 nm in a SynergyMx platereader (BioTek) that had been prewarmed to 37°C. The heat quench in this set up is relatively slow, and we found that it corresponded to the limit of the exponential fit in Figure 2C.

### TIRF Microtubule Elongation Assay

Experiments were conducted in flow cells (∼8 µL in volume) constructed using slides and silanized coverslips (Amersham Biosciences) attached with double-sided adhesive tape and bordered with vacuum grease. The flow cell was coated with 25% monoclonal anti-biotin (Clone BN-34, Sigma) and then blocked with 5% pluronic F-127 (Sigma). The chamber was then washed with 1 mg/mL casein in BRB80, and 6.25 µg/mL freshly thawed double-cycled GMPCPP (Jenna) [30] microtubule seeds were then introduced in the flow cell. These seeds were labeled 1∶1∶33 with biotin and Alexa488 (Cytoskeleton Inc). The chamber was then washed with 1 mg/mL casein in BRB80. Polymerization was initiated by introducing polymerization buffer (as described previously [31]) with 7.5 µM tubulin labeled 1∶50 with rhodamine tubulin (Cytoskeleton). The final buffer composition was BRB80 supplemented with 28 mM KCl and 3.75% glycerol (v/v). For the experiments with high concentrations of EB1, the final buffer composition was BRB80 supplemented with about 50 mM KCl and 5% glycerol. Imaging was performed at 37°C.

### TIRF Microtubule Nucleation Assay

Flow cells constructed as above were coated with 15% anti-tetraHis (Qiagen). The chamber was blocked with 5% pluronic F-127. p150 constructs were adsorbed to the chamber at 250 nM, and unbound constructs were removed with copious washing with BRB80 supplemented with 1 mg/mL BSA. Imaging was initiated and nucleation buffer perfused consisting of 3.5 µM tubulin in BRB80 supplemented with 1 mg/mL BSA, 2.5 mM GTP, 50 mM DTT, 140 mM glucose, glucose catalase/oxidase anti-fade system, and 0.25% F-127. Imaging was performed at 37°C.

### Microtubule Binding Assays

Unlabeled tubulin was polymerized at 5 mg/mL in BRB80 and 1 mM GTP and stabilized with 20 µM Taxol on the day of the experiment. Increasing concentrations of microtubules were incubated at 37°C for 20 min with 0.4 µM dimeric constructs or 0.8 µM monomeric construct, and centrifuged at 100 k rpm in the TLA 120.1 rotor (Beckman) at 37°C for 20 min. The supernatant and the pellet were then separated, denatured, and analyzed by SDS-PAGE and densitometry. For the binding assays done with C-terminal GFP-tagged constructs, microtubules were incubated with 0.1 µM constructs and the depletion of protein from the supernatant monitored by fluorimetry. Primary antibody for Western blotting was against the CAP-Gly domain of p150^Glued^ (BD Biosciences).

### Live Cell Imaging and Analysis

Isolation, culture, nucleofection, and imaging of DRG neurons was performed as done previously [19]. GFP-EB3 comets were imaged in both the mid- and distal axons, taking care to image only cells that were expressing at appropriately low levels. Kymographs were constructed and quantitated in ImageJ, using the Multiple Kymograph plugin (EMBL Heidelberg). The *x*-axis displacement of the comet we computed as the distance until catastrophe, while the slope of the line that the comet makes was taken as the polymerization velocity. COS7 cells were cultured, and transfected as described previously [16], and imaged as for neurons above. Cells expressing appropriate levels of GFP-EB3 were quantitated using the PlusTipTracker software package [40] using the following tracking parameters: search radius of 3–15 pixels; minimum subtrack length of three frames; maximum gap length of five frames; maximum shrinkage factor (relative to growth speed) of 1.5; maximum angle of 30 degrees forward and 10 degrees backward; and fluctuation radius of 1.0 pixels.

The sequences of the siRNA oligonucleotides against p150^Glued^ are GACUUCACCCCUUGAUUAA and CGAGCUCACCACAGACCUG. The corresponding scrambled sequences are GATCCTTTACGTCTCACAA and CCUACGCAAUCCGACCGAG, respectively [16],[60].

## Supporting Information

Dataset S1 **Analysis of nonmetazoan p150^Glued^ N-termini.** The Homo sapiens p150^Glued^ CAP-Gly was used as the query to perform a standard protein blast on the indicated organism. The results were then manually curated, and the core CAP-Gly domain in the listed sequence (highlighted in yellow) identified using the Conserved Domain Database. The p150^Glued^ CC1 was then identified using Coils (http://embnet.vital-it.ch/software/COILS_form.html, highlighted in magenta) and acidic and basic amino acids (red and blue respectively) highlighted using Protein Colourer (http://www.ebi.ac.uk/cgi-bin/proteincol/ProteinColourer.pl).
(DOCX)

Figure S1 **Characterization of recombinant dimeric and monomeric p150 polypeptides.** (A) Coomassie staining demonstrates purity of recombinant polypeptides used in experiments, and at right, dimerization of p150 Nt-GCN4 confirmed by glutaraldehyde cross-linking. (B) Hydrodynamic analysis of the p150 Nt-GCN4 and p150 Nt confirms monodispersion and suggests elongated shape. From left: mean elution volume ± SEM for at least three separate column runs; expected molecular mass from the primary amino acid sequence; apparent molecular mass from the calibrated column; calculated Stokes radius from the calibrated column; minimal Stokes radius of a perfectly spherical protein of the expected molecular mass; ratio of Rs/Rmin (Globular 1.2–1.3; moderately elongated 1.5–1.9; highly elongated >2.0) [61]. (C) Microtubule pelleting curves for 100 nM C-terminal GFP-tagged constructs incubated with increasing concentrations of Taxol-stabilized microtubules shows both constructs bind microtubules with moderate affinity. Percent binding was determined fluorimetrically from the fraction of construct in the pellet. Below, validation of fluorimetry by Western blotting the supernatants for p150. (D) Coomassie staining of recombinant polypeptides used in experiments. (E) Pellets of assembly reactions from Figure 1B confirm microtubule formation. (F) Light scattering traces for recombinant p150 proteins with and without tubulin confirm that measured turbidity does not result from protein aggregation at the elevated temperatures used in the assay. All error bars indicate the SEM for three or more independent experiments.
(TIF)

Figure S2 **Characterization of untagged p150 Nt-GCN4 or p150 Nt-GCN4-Strep.** (A) Coommassie-stained or immunloblot for His-tag demonstrate purity of recombinant proteins and confirm absence of tag. (B) Analytical size exclusion chromatography of p150 Nt-GCN4-His and corresponding untagged construct. (C) Light scattering traces for buffer control or recombinant polypeptides incubated with 20 µM tubulin and warmed to 37°C confirm that the ability of p150 to promote microtubule formation does not result from excess charge on the His-tag. Note that since they otherwise would be indistinguishable, the traces for the 1 µM His- or Strep-tagged p150 Nt-GCN4 alternate. (D) Light scattering traces for buffer control compared to varying concentrations of untagged p150 Nt-GCN4.
(TIF)

Figure S3 **p150^Glued^ modifies microtubule dynamics independently of EB1.** (A) Coomassie staining of purified full-length EB1 used in experiments. (B) Size exclusion chromatograms for EB1 and p150 Nt-GCN4 run alone, or pre-incubated in a 1∶2 ratio indicate that they form a stable complex. (C, D) Polymerization rates and catastrophe frequencies from seeded assembly shows that 200 nM p150 Nt-GCN4 promotes polymerization and inhibits catastrophe independently of 25 nM EB1; *p*<0.01 for all conditions compared to control. (E, F) Polymerization rates and catastrophe frequencies from seeded assembly for varying higher concentrations of EB1 with or without 200 nM p150 Nt-GCN4 confirm that p150 increases the microtubule polymerization rate independently of EB1 and damps the intrinsic pro-catastrophe activity of EB1. (G, H, I) Size exclusion chromatographs and corresponding fractions subjected to SDS-PAGE and coommassie staining suggest that at similar molar ratios, soluble p150 can complex with either tubulin or EB1.
(TIF)

Figure S4 **Characterization of complex formation between the p150 Nt and tuublin.** (A) Size exclusion chromatograms for p150 Nt run alone or pre-incubated with tubulin reveal that p150 forms a stable complex with tubulin. (B) Hydrodynamic analysis of the p150 Nt-GCN4 and p150 Nt complexes with tubulin suggests the formation of a globular complex. From left: mean elution volume ± SEM for at least three separate column runs; expected molecular mass from the primary amino acid sequence; apparent molecular mass from the calibrated column; calculated Stokes radius from the calibrated column; minimal Stokes radius of a perfectly spherical protein of the expected molecular mass; ratio of Rs/Rmin (Globular 1.2–1.3; moderately elongated 1.5–1.9; highly elongated >2.0) [61]. (C–I) Individual SEC fractions for the indicated conditions were subjected to SDS-PAGE and colloidal Coomassie staining. (J) Subtilisin was incubated in a 1∶350 weight ratio with tubulin at room temperature for the indicated times, then inactivated with 4 mM PMSF, and heat denatured. Twenty minutes cleavage was chosen for further experiments because separation of the β-tubulin band has plateaued and internal cleavage products have begun to form. (K) Size exclusion chromatograms of mock-treated tubulin or subtilisin-treated tubulin.
(TIF)

Figure S5 **Characterization of recombinant Δ5–7 Nt-GCN4 and p135 Nt-GCN4 representing endogenous isoforms.** (A) Coomassie staining of purified polypeptides used in experiments. (B) Coomassie stain of the recombinant dimers in the absence or presence of 0.5% glutaraldehyde demonstrates dimerization. (C) Size exclusion chromatograms of p150 Nt-GCN4, p150 Nt, Δ5–7 Nt-GCN4, and p135 Nt-GCN4 together to allow comparison. (D) Hydrodynamic analysis of the p150 polypeptides. From left: mean elution volume ± SEM for at least three separate column runs; expected molecular mass from the primary amino acid sequence; apparent molecular mass from the calibrated column; calculated Stokes radius from the calibrated column; minimal Stokes radius of a perfectly spherical protein of the expected molecular mass; ratio of Rs/Rmin (Globular 1.2–1.3; moderately elongated 1.5–1.9; highly elongated >2.0) [61]. (E, F) Size exclusion chromatograms for EB1 and Δ5–7 Nt-GCN4 or p135 Nt-GCN4 run alone or pre-incubated in a 1∶2 ratio indicate that Δ5–7 Nt-GCN4, which contains the CAP-Gly domain, but not p135 Nt-GCN4, which does not, can form a stable complex with EB1.
(TIF)

Figure S6 **Characterization of p150^Glued^ role in modulating microtubule dynamics in cells.** (A) Bioinformatics analysis of p150^Glued^ homologues in diverse metazoans characterizes the basicity downstream of the CAP-Gly domain. Using Homo sapiens p150^Glued^ as a search term, PSI-BLAST was performed to identify as many metazoan homologues as possible [62]. Then, full-length cDNA or, to avoid biases due to splicing, genomic DNA computationally spliced using GENSCAN [63], was pairwise aligned using Clustal Omega [64], and peptide characteristics computed using ExPASy ProtParam [65]. (B) Basicity versus divergence time plot indicates that over metazoan evolution, the region downstream of the p150^Glued^ CAP-Gly has evolved progressively more basicity. Divergence times calculated using TimeTree [66]. (C) GFP-EB3 comet distance to catastrophe was analyzed using kymograph analysis on a per-neuron basis to control for variable rescue transfection efficiency.
(TIF)

Figure S7 (A) Depiction of Q74P p150^Glued^ mutation using PyMol. (B, C) Hydrodynamic analysis of the p150 polypeptides shows that the mutant construct is similar to the wild-type construct. From left: mean elution volume ± SEM for at least three separate column runs; expected molecular mass from the primary amino acid sequence; apparent molecular mass from the calibrated column; calculated Stokes radius from the calibrated column; minimal Stokes radius of a perfectly spherical protein of the expected molecular mass; ratio of Rs/Rmin (Globular 1.2–1.3; moderately elongated 1.5–1.9; highly elongated >2.0) [61]. (D) Coomassie stain of the recombinant dimers in the absence or presence of 0.5% glutaraldehyde shows that the mutant construct is correctly dimerized.
(TIF)

## Abbreviations

+TIP: plus end-tracking protein
γ-TuRC: gamma-tubulin ring complex
CAP-Gly domain: cytoskeleton associated protein glycine-rich domain
CC: coiled-coil
DCX-GFP: doublecortin fused to GFP
DRG: dorsal root ganglion
EB1 or EB3: end-binding protein 1 or 3
GCN4: the dimerizing coiled-coil domain of the GCN4 protein
GMPCPP: guanylyl-(alpha, beta)-methylene-diphosphonate
MAP: microtubule-associated protein
MT: microtubule
Nt: amino-terminal
rKin430-GFP: first 430 amino acids of kinesin-1 fused to GFP
R_min_: minimum radius of a spherical protein with given molecular weight
R_s_: stokes radius
TIRF: total internal reflection fluorescence
