# Supplementary material for: Dynactin Subunit p150Glued Is a Neuron-Specific Anti-Catastrophe Factor
Source: PLoS Biol. 2013 Jul 16;11(7):e1001611. doi: 10.1371/journal.pbio.1001611 (PMC3712912; doi:10.1371/journal.pbio.1001611)
Supplement: Dataset S1 — Analysis of nonmetazoan p150Glued N-termini. The Homo sapiens p150Glued CAP-Gly was used as the query to perform a standard protein blast on the indicated organism. The results were then manually curated, and the core CAP-Gly domain in the listed sequence (highlighted in yellow) identified using the Conserved Domain Database. The p150Glued CC1 was then identified using Coils (http://embnet.vital-it.ch/software/COILS_form.html, highlighted in magenta) and acidic and basic amino acids (red and blue respectively) highlighted using Protein Colourer (http://www.ebi.ac.uk/cgi-bin/proteincol/ProteinColourer.pl). (DOCX) [file pbio.1001611.s001.docx]

**Supplemental dataset 1 –** Non-metazoan p150^Glued^ N-termini.

The Homo sapiens p150^Glued^ CAP-Gly was used as the query to perform a standard protein blast on the indicated organism. The results were then manually curated, and the core CAP-Gly domain in the listed sequence (highlighted in yellow) identified using the Conserved Domain Database. The p150^Glued^ CC1 was then identified using Coils (http://embnet.vital-it.ch/software/COILS_form.html, highlighted in magenta) and acidic and basic amino acids (red and blue respectively) highlighted using Protein Colourer (http://www.ebi.ac.uk/cgi-bin/proteincol/ProteinColourer.pl).

**>gi|13259510|ref|NP_004073.2| [Homo sapiens]**

MAQSKRHVYSRTPSGSRMSAEASARPLRVGSRVEVIGKGHRGTVAYVGATLFATGKWVGVILDEAKGKNDGTVQGRKYFTCDEGHGIFVRQSQIQVFEDGADTTSPETPDSSASKVLKREGTDTTAKTSKLRGLKPKKAPTARKTTTRRPKPTRPASTGVAGASSSLGPSGSASAGELSSSEPSTPAQTPLAAPIIPTPVLTSPGAVPPLPSPSKEEEGL

118 amino acids between CAP-Gly and CC1. 15 basic. 9 acidic.

**Protists**

>gi|118398234|ref|XP_001031446.1| [Tetrahymena thermophila]

MAESAFKVNDRIKLTNNANKDQEGTILYIGQLDGKEGIWIGVELDLPKGSHNGQFNGKQYFQGRDQHCMFVKEKHIQLIQQIKQPTPPKEKQQTEQPPQAEKPVEKKILTKPLGMGVGTKSTASTQGGGTGTTVLGPTAQKLIKNIQEKSATNQSKIPSSSQVQQQSAS

89 amino acids between CAP-Gly and CC1. 13 basic. 6 acidic.

>gi|145505621|ref|XP_001438777.1| [Paramecium tetraurelia]

MEQLQEGDYVTILKDPIKNEKGYIRYLGELEGRPGTFYGIHLDNKVGSHNGTLQGKEYFTCPEGHGLFITGNHLKKTTMVTRDPNKKSTKVTKDEPKPQKLKTSASSAAVVQKAQPQQQSQYGSNQTTKQTTLKRNESKK

64 amino acids between CAP-Gly and CC1. 12 basic. 3 acidic.

**Filamentous fungi**

>gi|67540306|ref|XP_663927.1| [Aspergillus nidulans]

MAELTIGSVIALTDGRQATVRFIGATSFADGEWIGVELTDDTGKNDGSVQGERYFDCEPGFGMFVRPTAVASTPSKSPTKQLTSATPASRPSISGSSRPSVAAPKPRPTTTKPSMGPPTQSTTSRAARTSLSGPGNKTNRQSLQGTAGTTSSGLSKRPTLRPTPTTRTSEEPSPATEGSETLSNDVEGDIEEDLGPQPAHPARTSSSRLLSGQSASPRQSQNMA

147 amino acids between CAP-Gly and CC1. 17 basic. 10 acidic.

>gi|58266192|ref|XP_570252.1| [Cryptococcus neoformans]

MTSQEVPIDAKVQVSAGIGYVRWTGANPGFAAGKWVGVELFEAGGKNDGSVKGERYFECKPNHGVFVRPSQVRILEAPMPTETPRPQSTRPSATPSAHRLTSASSARAASPQKQPTTRSTAPPTPSQTLRVVSGSSSIEDVVPSTAPMTRRVSNSSSSRPSQGIFKRPPSVLDARALTIATEDIEHVAAHSIVSPPPGRISSPTRRVVSPTPSISQQSRTRSISSAFVAPPDPAALEQASTQPQSLSRPPSPDEEHT

177 amino acids between CAP-Gly and CC1. 19 basic. 10 acidic.

>gi|164427433|ref|XP_955770.2| [Neurospora crassa]

MSELGVAVGQKIELADGSGRTAFVRYVGETAFAPGTWVGIELDEPSGKNDGSVQGERYFNCEMGYGMFVRPTTFNVIAQPPPPPPPSTFRRSVTTRPTSLNASTTRRPAPVDSGLAKRMSLNAPSPSPGPRPSRTSSTSITRSPTRSPTKQLATASSSGNPSRSGTPSTTTKPAGPTTRTRPSLSTSRHSMGPPPTPTTRTTRKPSVSSVGTRPSIGATRPVGGRASMSARSSTNRLSDPRESTGSVSSVGKSGFKRGSASPRSSDEELSASPVPASPVHQKTAALEKLTAPGAGNGGGGASPGATSPNLKATTITPRSSITNT

245 amino acids between CAP-Gly and CC1. 33 basic. 7 acidic.

>gi|71019485|ref|XP_759973.1| [Ustilago maydis]

MSTPRAPGTAPQPIHLHSRVKVSNLGHGEVLFVGQTSFAPGVWVGIELDEQNGKNNGSVQGKRYFECDDGYGVFVRSSQVHVLSPEEEMHSVDDESMPPPPTPSSTVTRPAATLTPSAAAAARLSPRKSVAPTPSRAPATRSSLAPAAPPSATRRTSATSVSSSISSASASRPPTSASITSRTSSPIKPSTGVRSPVKPTATGRISAASSMMRSAAAASSLANKTASPSTPVATARTLRTANTPASAALSSARTPTAGSGMTRTAQRAIPSAVSASAKPQTPAAARAGLRPVPASRPSMISGASRTAASSPSVASTSRVGAARAAAASAPISRAASSISIVSRAGSSLGPSRLGPRSSAGSAVSSGRTSASRRLQDPDDDDADYLLGAQDDDQDDLLATESYANLAQTDEDNVVDDADQTIDAGSAADSTLRSTKLLNKSTRDFMSLVEPSSSNAAVRS

339 amino acids between CAP-Gly and CC1. 39 basic. 28 acidic.

**Yeasts**

>gi|6325083|ref|NP_015151.1| [Saccharomyces cerevisiae]

MRNAGVQVDTNMQKISLQDTVLVNEMKGRVKFIGETQFAKGIWYGIELDKPLGKNDGSANGIRYFDIDLKKANSNGGYYGLFCKKDTLQFYKPDDDEHSLLNGNAAQ

10 amino acids between CAP-Gly and CC1. 1 basic. 4 acidic.

>gi|19115531|ref|NP_594619.1| [Schizosaccharomyces pombe]

MSYLSVGDEVLIRGELGIVRFAGSTDFESGIWLGVELLNGKGKNDGSVKGKRYFSCEKGKGIFVRASSNVMKRPSVVKSRKKGSENISNFMEKTKAIKQKSRREPSKFERSLARPLCITPIDSSTPTKTATFYTSSTTENLDELNFSTEELSSFDTTLLNSDTSKLSGLDDSSFMEEEFVWQVDNVLQECEKKFTPHSKGSYLKENLKSELRKGR

144 amino acids between CAP-Gly and CC1. 22 basic. 21 acidic.
