# Supplementary figures and images for: Dynactin Subunit p150Glued Is a Neuron-Specific Anti-Catastrophe Factor
Source: PLoS Biol. 2013 Jul 16;11(7):e1001611. doi: 10.1371/journal.pbio.1001611 (PMC3712912; doi:10.1371/journal.pbio.1001611)

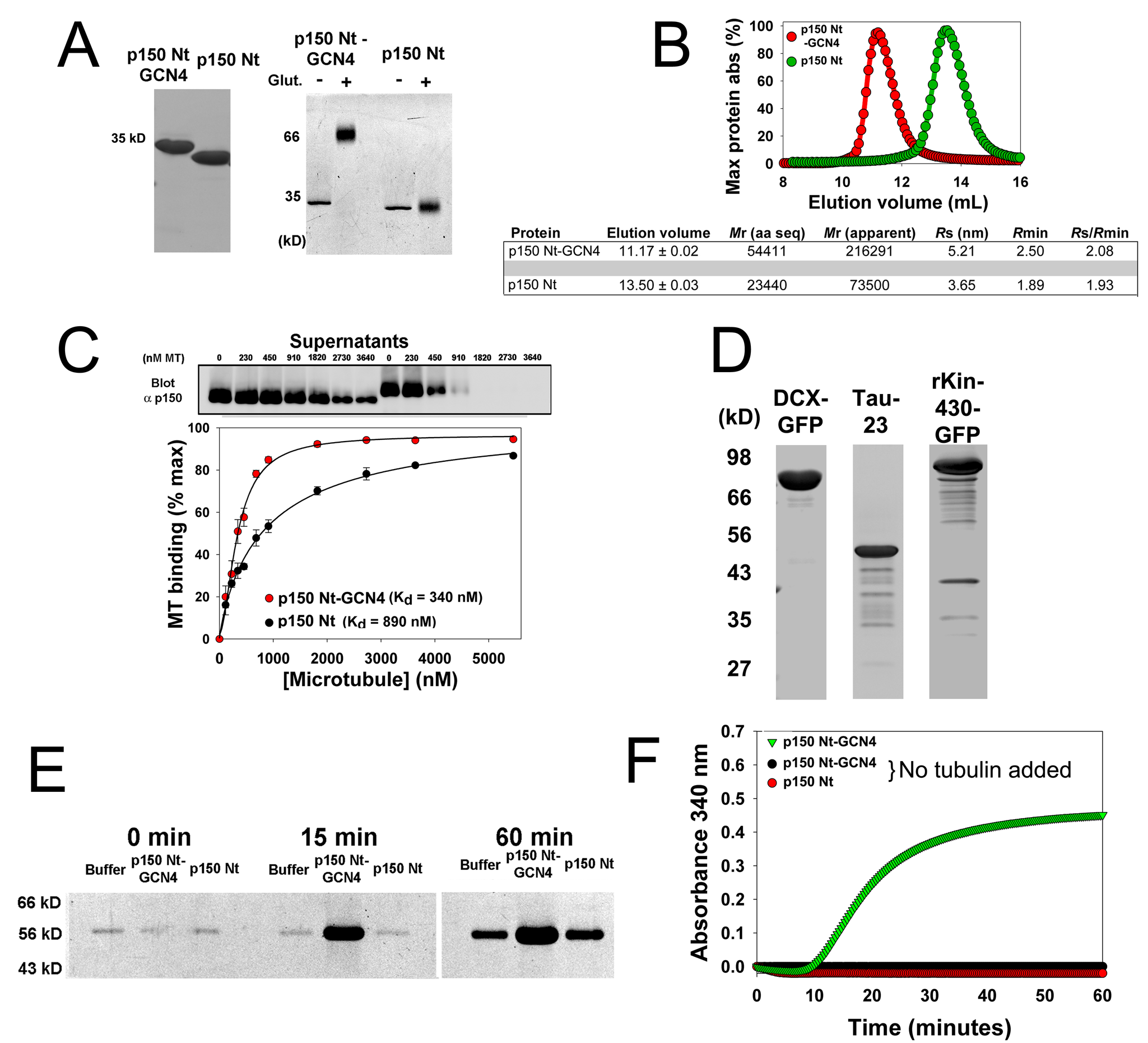

Supplement: Figure S1 — Characterization of recombinant dimeric and monomeric p150 polypeptides. (A) Coomassie staining demonstrates purity of recombinant polypeptides used in experiments, and at right, dimerization of p150 Nt-GCN4 confirmed by glutaraldehyde cross-linking. (B) Hydrodynamic analysis of the p150 Nt-GCN4 and p150 Nt confirms monodispersion and suggests elongated shape. From left: mean elution volume ± SEM for at least three separate column runs; expected molecular mass from the primary amino acid sequence; apparent molecular mass from the calibrated column; calculated Stokes radius from the calibrated column; minimal Stokes radius of a perfectly spherical protein of the expected molecular mass; ratio of Rs/Rmin (Globular 1.2–1.3; moderately elongated 1.5–1.9; highly elongated >2.0) [61]. (C) Microtubule pelleting curves for 100 nM C-terminal GFP-tagged constructs incubated with increasing concentrations of Taxol-stabilized microtubules shows both constructs bind microtubules with moderate affinity. Percent binding was determined fluorimetrically from the fraction of construct in the pellet. Below, validation of fluorimetry by Western blotting the supernatants for p150. (D) Coomassie staining of recombinant polypeptides used in experiments. (E) Pellets of assembly reactions from Figure 1B confirm microtubule formation. (F) Light scattering traces for recombinant p150 proteins with and without tubulin confirm that measured turbidity does not result from protein aggregation at the elevated temperatures used in the assay. All error bars indicate the SEM for three or more independent experiments. (TIF) [file pbio.1001611.s002.tif]

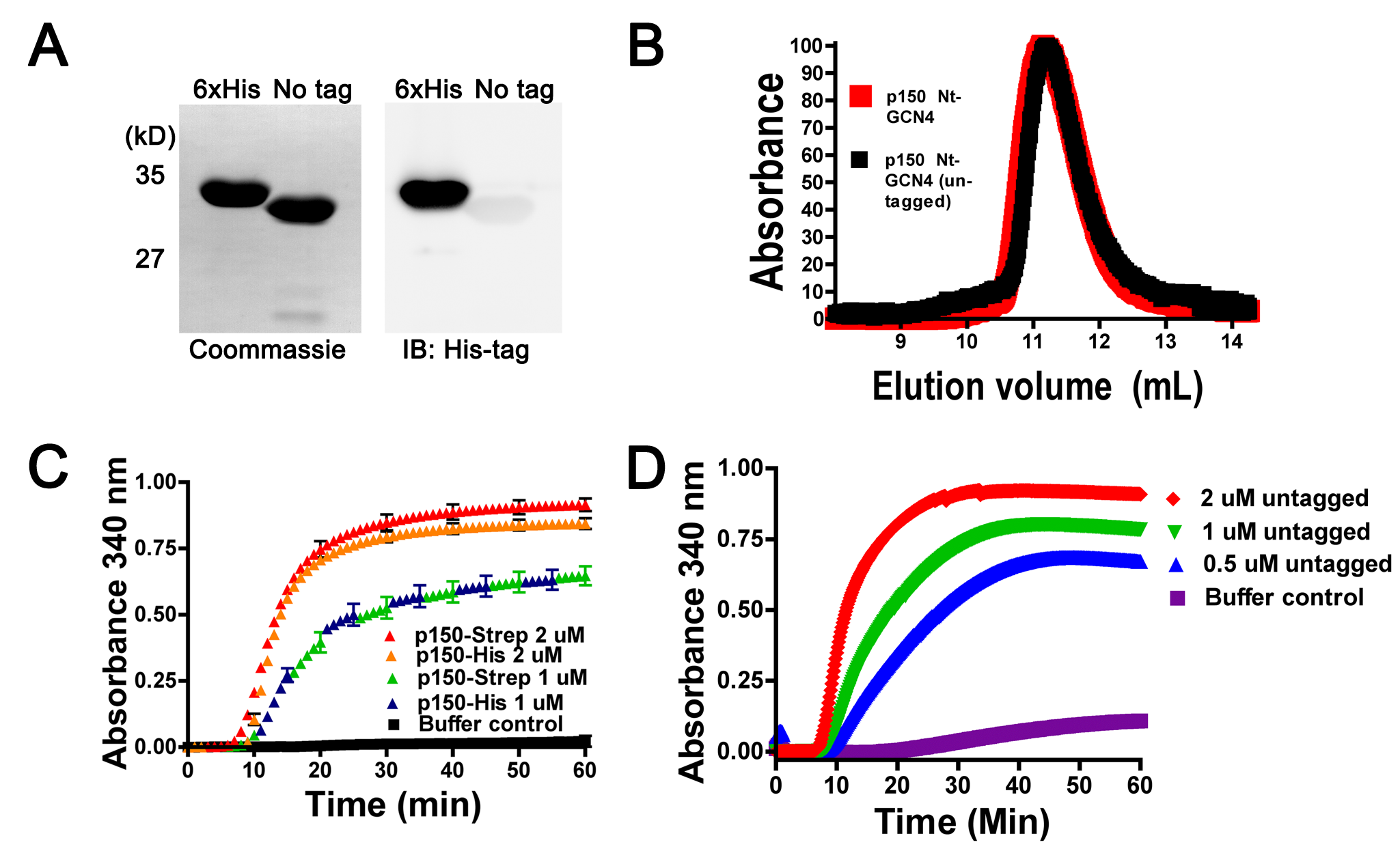

Supplement: Figure S2 — Characterization of untagged p150 Nt-GCN4 or p150 Nt-GCN4-Strep. (A) Coommassie-stained or immunloblot for His-tag demonstrate purity of recombinant proteins and confirm absence of tag. (B) Analytical size exclusion chromatography of p150 Nt-GCN4-His and corresponding untagged construct. (C) Light scattering traces for buffer control or recombinant polypeptides incubated with 20 µM tubulin and warmed to 37°C confirm that the ability of p150 to promote microtubule formation does not result from excess charge on the His-tag. Note that since they otherwise would be indistinguishable, the traces for the 1 µM His- or Strep-tagged p150 Nt-GCN4 alternate. (D) Light scattering traces for buffer control compared to varying concentrations of untagged p150 Nt-GCN4. (TIF) [file pbio.1001611.s003.tif]

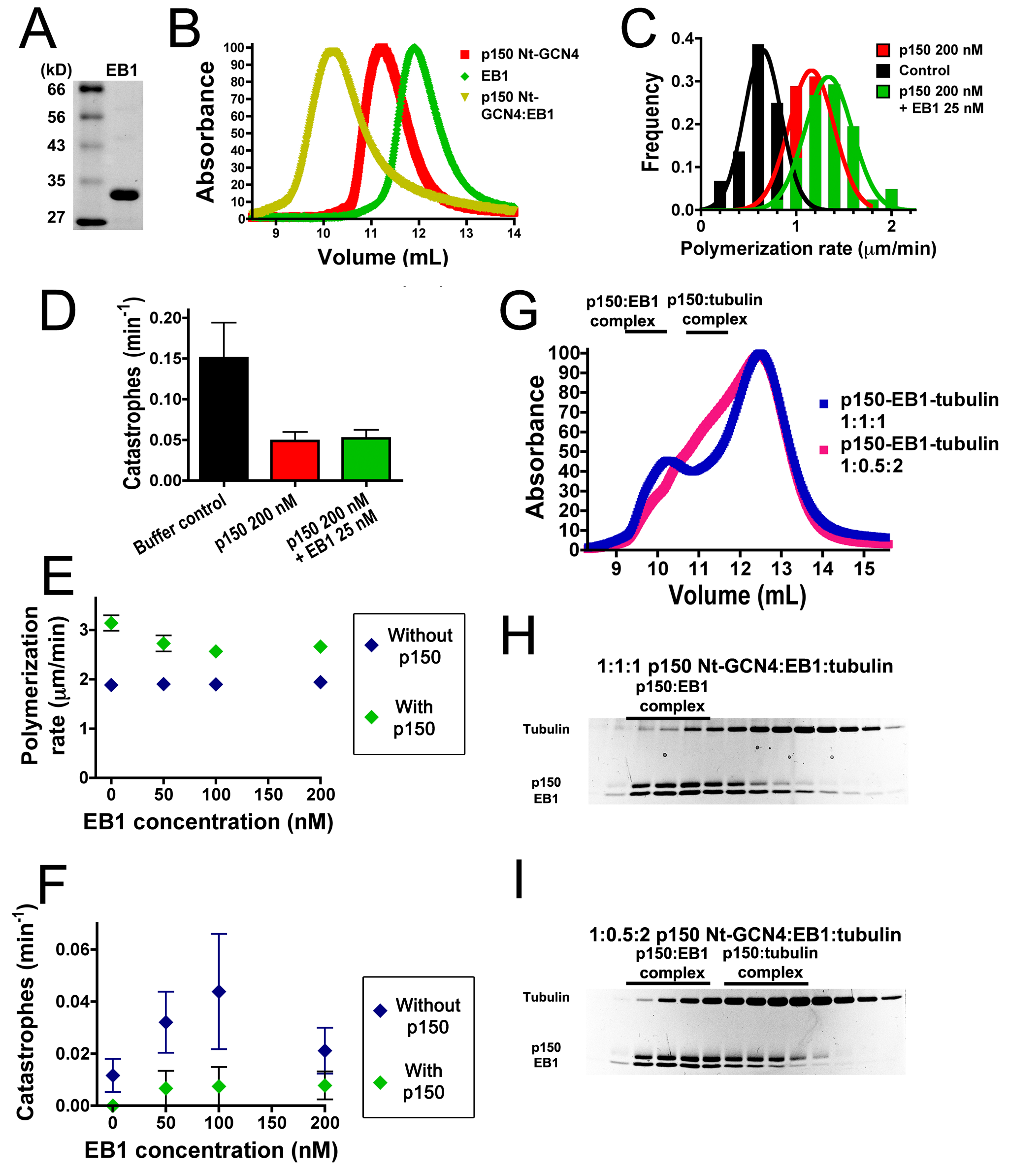

Supplement: Figure S3 — p150Glued modifies microtubule dynamics independently of EB1. (A) Coomassie staining of purified full-length EB1 used in experiments. (B) Size exclusion chromatograms for EB1 and p150 Nt-GCN4 run alone, or pre-incubated in a 1∶2 ratio indicate that they form a stable complex. (C, D) Polymerization rates and catastrophe frequencies from seeded assembly shows that 200 nM p150 Nt-GCN4 promotes polymerization and inhibits catastrophe independently of 25 nM EB1; p<0.01 for all conditions compared to control. (E, F) Polymerization rates and catastrophe frequencies from seeded assembly for varying higher concentrations of EB1 with or without 200 nM p150 Nt-GCN4 confirm that p150 increases the microtubule polymerization rate independently of EB1 and damps the intrinsic pro-catastrophe activity of EB1. (G, H, I) Size exclusion chromatographs and corresponding fractions subjected to SDS-PAGE and coommassie staining suggest that at similar molar ratios, soluble p150 can complex with either tubulin or EB1. (TIF) [file pbio.1001611.s004.tif]

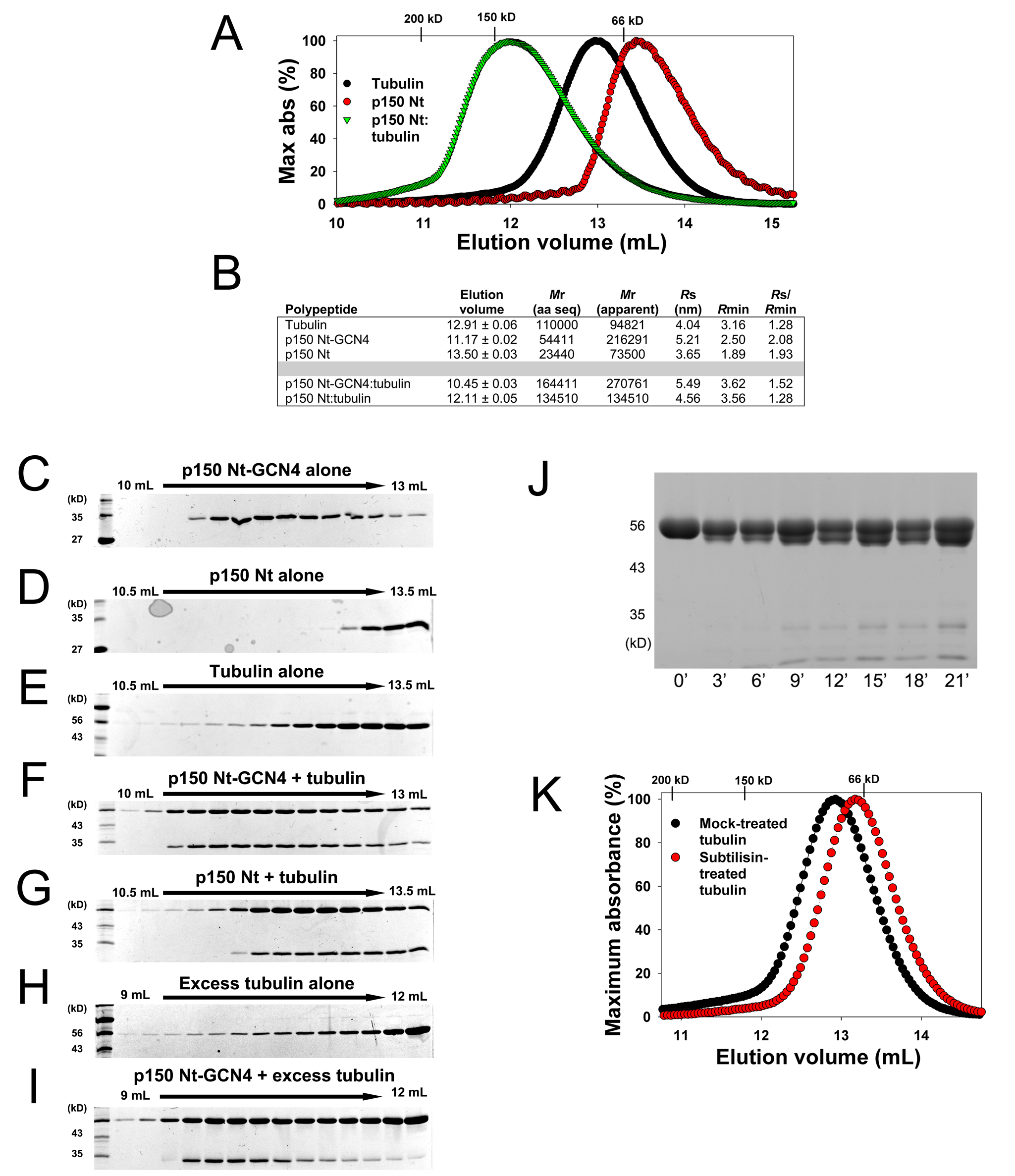

Supplement: Figure S4 — Characterization of complex formation between the p150 Nt and tuublin. (A) Size exclusion chromatograms for p150 Nt run alone or pre-incubated with tubulin reveal that p150 forms a stable complex with tubulin. (B) Hydrodynamic analysis of the p150 Nt-GCN4 and p150 Nt complexes with tubulin suggests the formation of a globular complex. From left: mean elution volume ± SEM for at least three separate column runs; expected molecular mass from the primary amino acid sequence; apparent molecular mass from the calibrated column; calculated Stokes radius from the calibrated column; minimal Stokes radius of a perfectly spherical protein of the expected molecular mass; ratio of Rs/Rmin (Globular 1.2–1.3; moderately elongated 1.5–1.9; highly elongated >2.0) [61]. (C–I) Individual SEC fractions for the indicated conditions were subjected to SDS-PAGE and colloidal Coomassie staining. (J) Subtilisin was incubated in a 1∶350 weight ratio with tubulin at room temperature for the indicated times, then inactivated with 4 mM PMSF, and heat denatured. Twenty minutes cleavage was chosen for further experiments because separation of the β-tubulin band has plateaued and internal cleavage products have begun to form. (K) Size exclusion chromatograms of mock-treated tubulin or subtilisin-treated tubulin. (TIF) [file pbio.1001611.s005.tif]

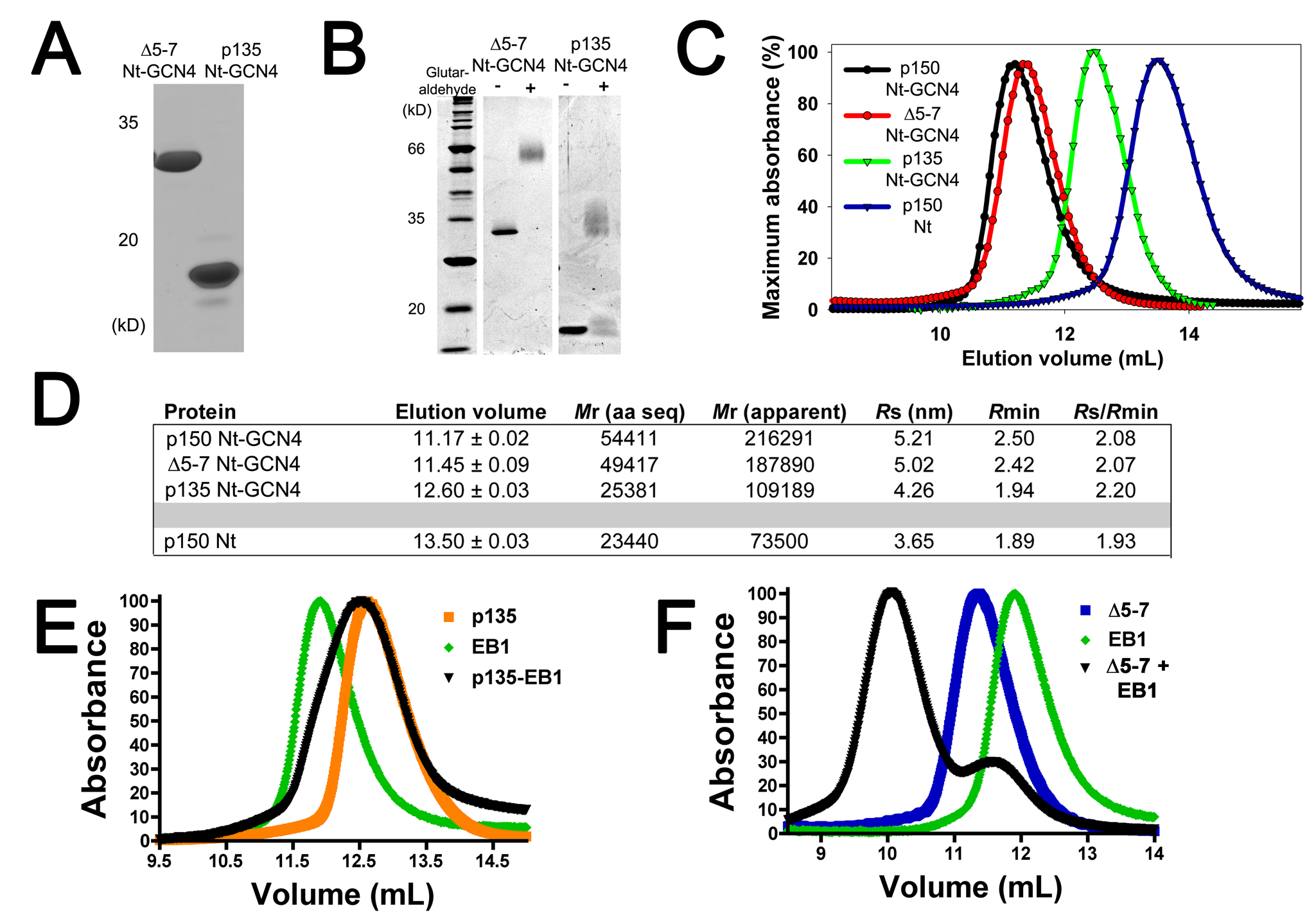

Supplement: Figure S5 — Characterization of recombinant Δ5–7 Nt-GCN4 and p135 Nt-GCN4 representing endogenous isoforms. (A) Coomassie staining of purified polypeptides used in experiments. (B) Coomassie stain of the recombinant dimers in the absence or presence of 0.5% glutaraldehyde demonstrates dimerization. (C) Size exclusion chromatograms of p150 Nt-GCN4, p150 Nt, Δ5–7 Nt-GCN4, and p135 Nt-GCN4 together to allow comparison. (D) Hydrodynamic analysis of the p150 polypeptides. From left: mean elution volume ± SEM for at least three separate column runs; expected molecular mass from the primary amino acid sequence; apparent molecular mass from the calibrated column; calculated Stokes radius from the calibrated column; minimal Stokes radius of a perfectly spherical protein of the expected molecular mass; ratio of Rs/Rmin (Globular 1.2–1.3; moderately elongated 1.5–1.9; highly elongated >2.0) [61]. (E, F) Size exclusion chromatograms for EB1 and Δ5–7 Nt-GCN4 or p135 Nt-GCN4 run alone or pre-incubated in a 1∶2 ratio indicate that Δ5–7 Nt-GCN4, which contains the CAP-Gly domain, but not p135 Nt-GCN4, which does not, can form a stable complex with EB1. (TIF) [file pbio.1001611.s006.tif]

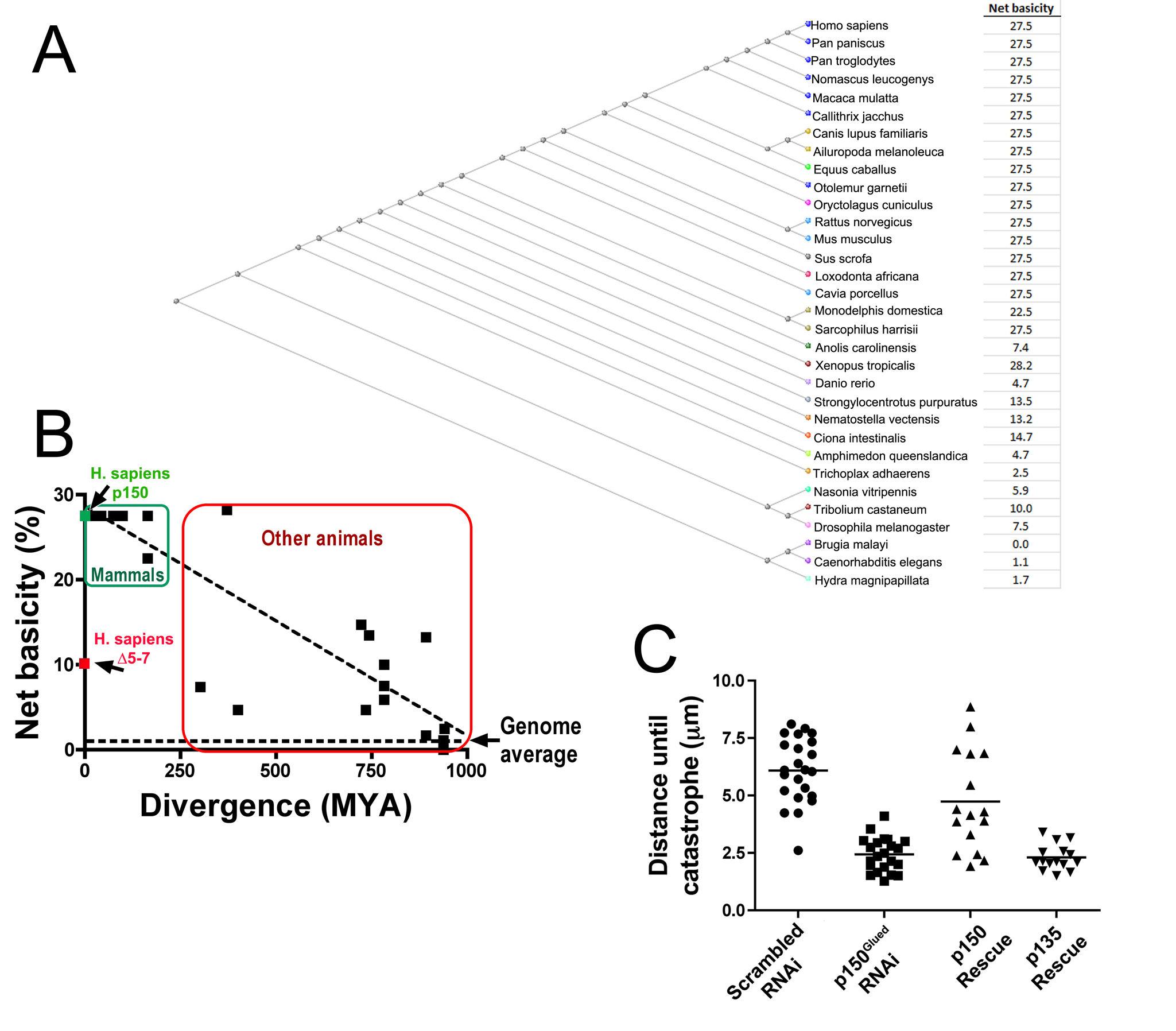

Supplement: Figure S6 — Characterization of p150Glued role in modulating microtubule dynamics in cells. (A) Bioinformatics analysis of p150Glued homologues in diverse metazoans characterizes the basicity downstream of the CAP-Gly domain. Using Homo sapiens p150Glued as a search term, PSI-BLAST was performed to identify as many metazoan homologues as possible [62]. Then, full-length cDNA or, to avoid biases due to splicing, genomic DNA computationally spliced using GENSCAN [63], was pairwise aligned using Clustal Omega [64], and peptide characteristics computed using ExPASy ProtParam [65]. (B) Basicity versus divergence time plot indicates that over metazoan evolution, the region downstream of the p150Glued CAP-Gly has evolved progressively more basicity. Divergence times calculated using TimeTree [66]. (C) GFP-EB3 comet distance to catastrophe was analyzed using kymograph analysis on a per-neuron basis to control for variable rescue transfection efficiency. (TIF) [file pbio.1001611.s007.tif]

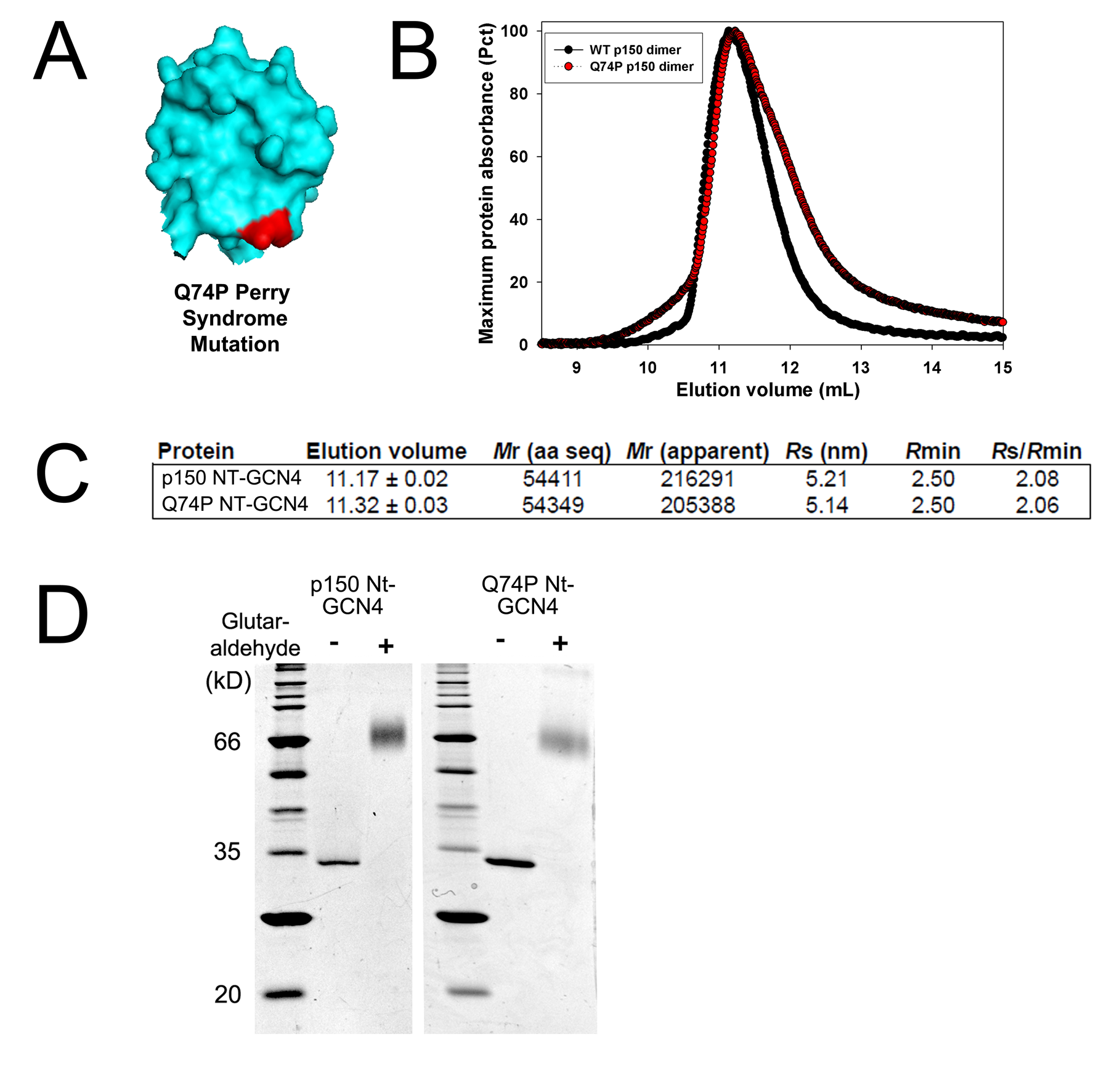

Supplement: Figure S7 — (A) Depiction of Q74P p150Glued mutation using PyMol. (B, C) Hydrodynamic analysis of the p150 polypeptides shows that the mutant construct is similar to the wild-type construct. From left: mean elution volume ± SEM for at least three separate column runs; expected molecular mass from the primary amino acid sequence; apparent molecular mass from the calibrated column; calculated Stokes radius from the calibrated column; minimal Stokes radius of a perfectly spherical protein of the expected molecular mass; ratio of Rs/Rmin (Globular 1.2–1.3; moderately elongated 1.5–1.9; highly elongated >2.0) [61]. (D) Coomassie stain of the recombinant dimers in the absence or presence of 0.5% glutaraldehyde shows that the mutant construct is correctly dimerized. (TIF) [file pbio.1001611.s008.tif]
